# Cryptococcal Antigenemia In South African Children Living With HIV

**DOI:** 10.1097/INF.0000000000005154

**Published:** 2026-01-28

**Authors:** Alison Gifford, Rudzani Mashau, Ruth Mpembe, David Khanyile, Thabo Maota, Mbali Dube, Boitumelo Kgoale, Adilia Warris, Nelesh P. Govender

**Affiliations:** 1 MRC Centre for Medical Mycology, Dept of Biosciences, Faculty of Life and Health Sciences, University of Exeter, UK; 2 National Institute for Communicable Diseases (NICD), a Division of the National Health Laboratory Service, Johannesburg, South Africa; 3 Wits Mycology Division, School of Pathology, University of the Witwatersrand, Johannesburg, South Africa; 4 Epicentre Health Research, Johannesburg, South Africa

## Introduction

Cryptococcal meningitis (CM) is a fungal infection of the brain which affects an estimated 200,000 people/year, predominantly those with immunosuppression due to Human Immunodeficiency Virus (HIV) infection.^1, 2^ It is almost uniformly fatal if untreated and accounts for 19% of AIDS-related deaths in adults^1^. The World Health Organization (WHO) recommends cryptococcal antigen (CrAg) screening in adults and adolescents (>10 years old) living with HIV with a CD4 count <200 cells/µL as pre-emptive treatment with fluconazole reduces mortality in adults.^3–5^ The WHO also recommends a lumbar puncture (LP) for those with a positive CrAg screening test, even if asymptomatic, to investigate subclinical CM.^4^ No recommendation is provided for children less than 10 years of age as CM incidence is presumed to be very low based on the scarce data available. In countries with a high burden of HIV, however, pediatric CM is comparable in incidence to other causes of pediatric meningitis such as *Haemophilus influenzae*.^6, 7^ Moreover, it is associated with a high mortality and neurodevelopmental sequelae.^8–12^

South Africa has one of the highest prevalences of HIV globally at 17.1% of the general population,^13^ and a high incidence of CM at 60 cases per 100,000 people living with HIV.^14^ In 2023 there were 160,000 children 0-14 years of age living with HIV in South Africa, with only 63% receiving antiretroviral treatment (ART)^13^ and a high prevalence of severe immunosuppression.^15^ The WHO considers any child under five years old living with HIV to have advanced HIV disease (AHD) due to their increased risk of rapid HIV progression and opportunistic infections. CD4 percentage, which remains stable in this age group, is often used due to the normal age-related decline in absolute CD4 count.^16, 17^ Children five years and over are considered to have AHD if their absolute CD4 count is <200 cells/µL.^18^ The South African National Health Laboratory Service (NHLS) implemented a national reflex CrAg screening program targeted at persons living with AHD in October 2016, in which any plasma sample with a CD4 count of <100 cells/µL (regardless of patient age) automatically had a CrAg test added without the need of a clinician request.^19^ This reflex screening method means that a CrAg test is repeated whenever a patient has a CD4 result <100 cells/µL. Only the first CrAg-positive result *or* a CrAg-positive result with signs of meningitis should prompt an LP and pre-emptive fluconazole.^20^

The prevalence of cryptococcal antigenemia in South African adults with CD4 count of <100 cells/µL is 5.8%^21^. The prevalence in South African children less than 18 years old is unknown. A reflex CrAg study from KwaZulu-Natal province showed that the CrAg prevalence between June 2015 and July 2016 was 3.5% (40/1126) in children and adolescents less than 20 years old compared to 5.8% (1194/20475) in adults.^22^ In Cameroon, the prevalence of cryptococcal antigenemia in children less than 16 years old living with HIV was 6.1%, although clear inclusion criteria were not provided^23^.

Meiring et al^8^, who described the incidence of pediatric cryptococcosis in 2005-2007, stated that “little is known about why immunocompromised children develop the disease less frequently than immunocompromised adults.” The 2018 WHO recommendation argues that the low incidence reflects a lack of exposure to *Cryptococcus*.^24^ However, most children over two years of age had been exposed as shown in a USA-based seroprevalence study.^25^ The low incidence may instead be an artefact as incidence is not consistent across the childhood years, showing bimodal peaks in infants and adolescents.^8^

The aim of this study is to describe the prevalence and clinical management of cryptococcal antigenemia in South African children. We hypothesized that the prevalence of antigenemia is sufficiently high in children and adolescents to justify targeted screening, and that management of children with antigenemia is poor owing to a lack of focus on this intervention in this population.

## Methods

### Patient population

All children and adolescents less than 18 years of age with a CD4 count of < 100 cells/µl and a reflex CrAg blood test result between January 2017 and December 2022 were identified from NHLS records in South Africa. The NHLS serves all public-sector facilities in South Africa. Any result with a missing date of birth (DOB) was excluded. Data accompanying the CrAg result including the CD4 absolute count, CD4 percentage and requesting healthcare facility were also extracted. Exact duplicate entries were removed to give the total number of positive and negative reflex CrAg results. Cryptococcal antigenemia prevalence was calculated by removing repeated tests on individual patients using probabilistic data linkage with pre-defined NHLS criteria^26^ and RecordLinkage in R v4.2.2. The first result for each patient was retained, except when a child initially tested CrAg-negative and later tested CrAg-positive, in which case only the first CrAg-positive result was included.

### Medical record review

Fifteen healthcare facilities in the Gauteng province were chosen for a convenience sample of CrAg-positive children to collect detailed clinical information. These included tertiary referral hospitals, district hospitals and community health clinics. For each CrAg-positive child, a CrAg-negative control was identified from the same healthcare facility and individually matched on age, sex and CD4 count; another matched control was identified if the medical records for the first were missing. All available medical records of these CrAg-positive children and CrAg-negative controls were imaged using CamScanner with patient identifiers concealed. Multiple visits to each facility were performed and wards and mortuaries searched for missing records. NHLS online laboratory results were checked to gather additional microbiology results potentially missing from the paper medical records. Clinical data were abstracted into the RedCap database including demographics, symptoms, neurological examination, CD4 counts, cerebrospinal fluid (CSF) laboratory results, pre-emptive fluconazole therapy or CM treatment and outcomes including subsequently developing CM during the 12-months after the reflex CrAg test, and mortality. Patients were grouped into those with meningitis symptoms at the time of the reflex CrAg test, those documented as asymptomatic and those with no documented symptoms. Children who had an episode of CM treated prior to the study period and who were now asymptomatic but had a residual positive blood CrAg test were excluded from further analysis. It is known that blood CrAg can remain positive after successful treatment and no further investigations are clinically warranted unless new symptoms of meningitis occur.^20^

Data analysis was performed using R version 4.2.2 (R Foundation for Statistical Computing, Vienna, Austria). The Mann Whitney, chi-squared or Fisher’s exact test was used to compare groups by age, CD4 count and percentage, CrAg result and antiretroviral treatment. The median and interquartile range (IQR) are shown unless stated otherwise.

### Ethics

Ethical approval was gained through the University of Witwatersrand, Johannesburg, MED23-05-016. Provincial approval for Gauteng (GP 202305 018) and individual facility approvals were also obtained.

## Results

### Cryptococcal antigenemia screening results in South African children

**Figure 1:**
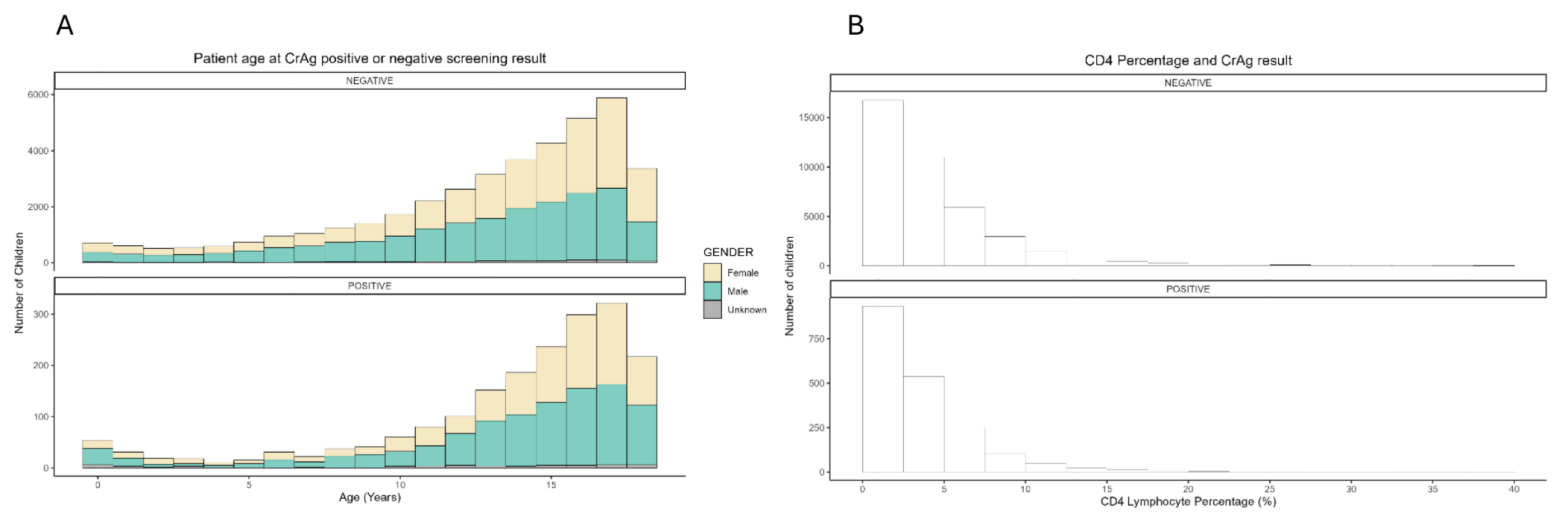
(a) Number of blood reflex cryptococcal antigen (CrAg) positive or negative test results between Jan 2017 and Dec 2022 shown by age (years) and documented gender. (b) CD4 lymphocyte percentage at the time of the blood reflex CrAg test shown by positive and negative results.

There were 2,483,726 reflex CrAg screening tests performed in South Africa in 2017-2022, of which 42,369 (1.7%) had an accompanying DOB confirming an age <18 years old. Of those performed in children, 4.6% (1933/42,369) tests were positive (Fig. 1a). In this pediatric age group, the male to female ratio for CrAg-positive results was 1.7:1 and for CrAg-negative results 1:1. The median age for children with CrAg-positive results was 15.0 years (IQR, 12.1-16.7) and with CrAg-negative results was 14.1 years (IQR, 10.5-16.3; p<0.001). The median CD4 percentage in children with CrAg-positive results was 2.6% (IQR, 1.2%-4.8%) and for negative results was 3.1% (IQR, 1.4%-5.9%; p<0.001; see Fig. 1b).

There were 1111 CrAg tests performed in infants less than one year of age, of which 68 (6.1%) were positive. The median CD4 percentage for these CrAg-positive infants was 4.8% (IQR, 2.8%-6.7%), with 95% having a CD4 percentage <15%. Of CrAg tests in children 1-4 years old, 3.1% (72/2326) were CrAg-positive, with a median CD4 percentage for these CrAg-positive children of 3.9% (IQR, 1.5%-6.2%); 96% of them had a CD4 percentage <15%. Of children 5-9 years old, 2.8% (173/6030) of CrAg tests were positive, with the CrAg-positive children having a median absolute CD4 count of 28 cells/µL (IQR, 11-58).

### Cryptococcal antigenemia prevalence in South African children

The CrAg-positive prevalence was 4.7% (1352/28,839) in children <18 years of age with a CD4 <100 cells/µl. In children <10 years old, for whom the WHO does not currently recommend screening, the CrAg-positive prevalence was 3.5% (261/7,440).

**Figure 2:**
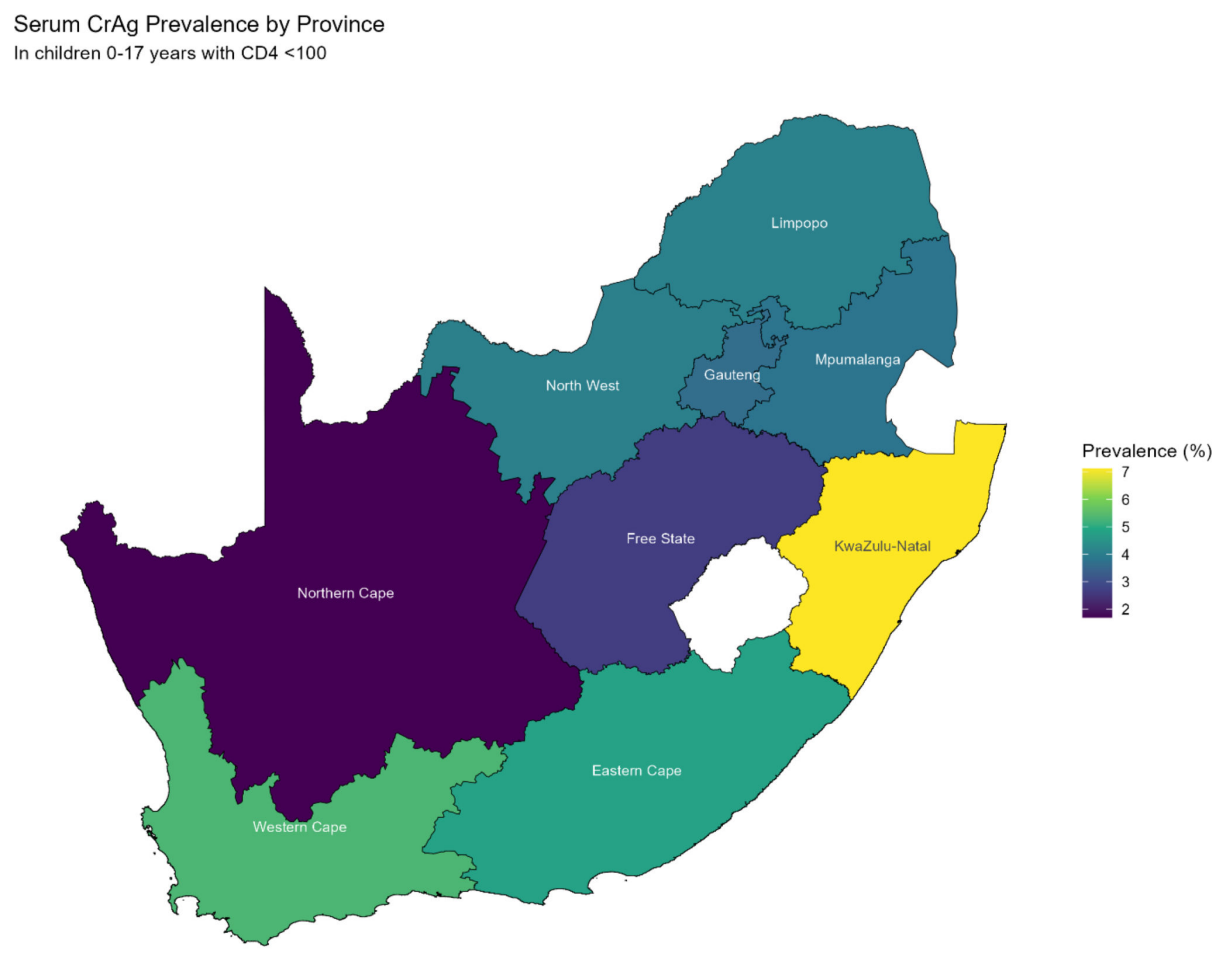
Cryptococcal antigenemia prevalence in children <18 years with a CD4 count <100cells/µl per province in South Africa. Repeated testing in individuals was removed and prevalence calculated as number of CrAg-positive children divided by total number of children with a CrAg result.

There was geographical variation. KwaZulu-Natal had the highest prevalence: 7.1% (498/6,996) in 0–17-year-olds and 4.6% (75/1,620) in 0–9-year-olds (Fig. 2). The median age in KwaZulu-Natal (14 years [IQR 12-16] vs. 14 years [IQR 11-16]; p=0.08) was not different from the overall pediatric population living with HIV and the CD4 count was slightly higher (27 [IQR 11-57] vs 23 [IQR 9-54]; p = 0.02).

### Clinical presentation of CrAg positive children

**Figure 3:**
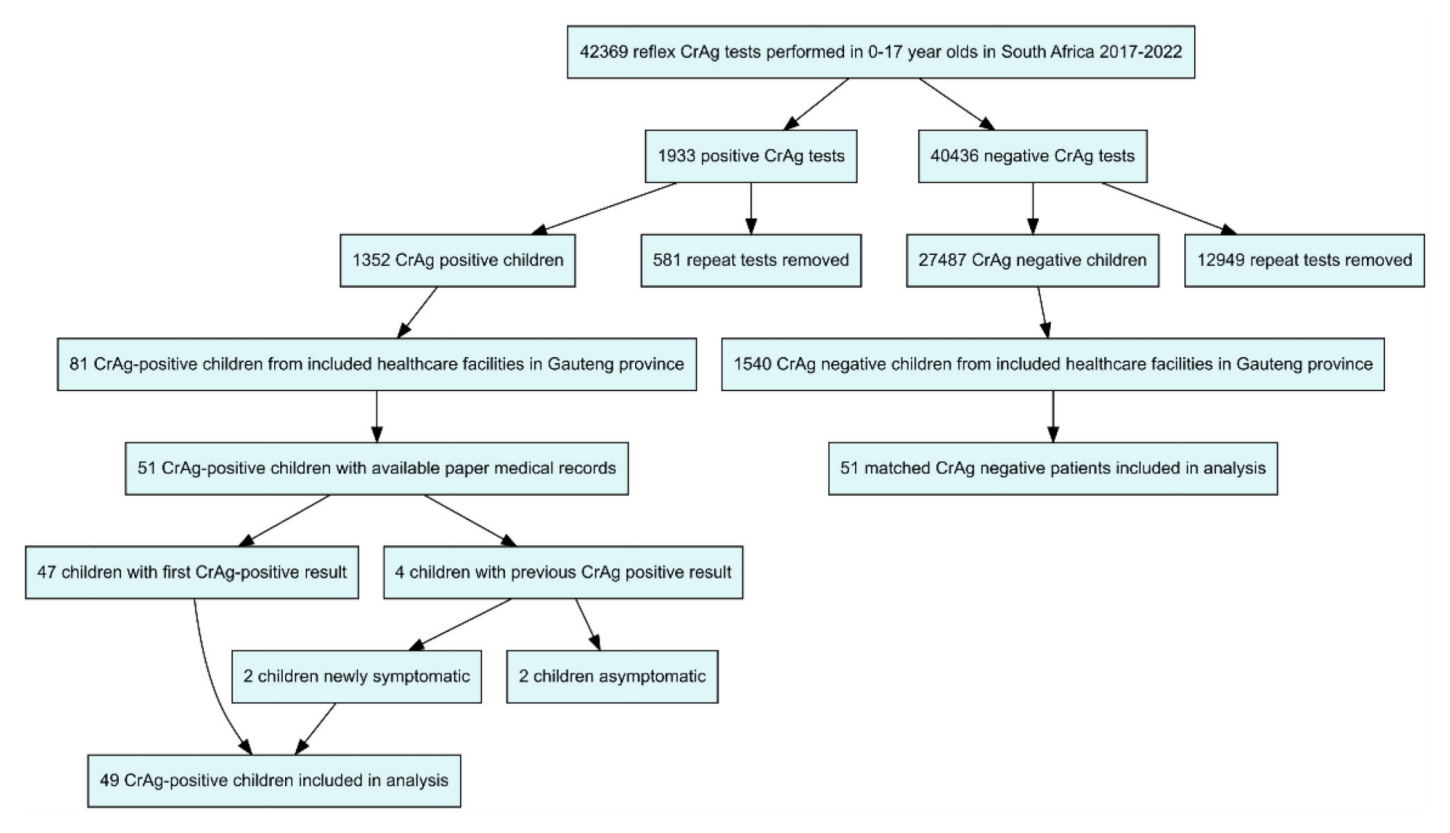
Flow diagram of patients included in retrospective medical record review. Cryptococcal antigen (CrAg) test results obtained from the National Health Laboratory Service records. Repeat tests in individuals identified by probabilistic data linkage and removed.

Eighty-one children with positive CrAg results were treated across 15 health care facilities in the Gauteng province. Medical records from 63% (51/81) could be located, combined with an equal number of age, sex and CD4 count matched CrAg-negative controls (Figure 3 and Table 1). Only 71% (36/51) of CrAg-positive and 33% (17/51; p<0.001) of CrAg-negative results were documented in the medical records. For 47 of the CrAg-positive children (92%), this was their first CrAg-positive result. Four had had a positive CrAg-test prior to the study period, of which two presented with new symptoms of meningitis. The remaining two had previously been treated for CM and were asymptomatic during the study period, so were therefore excluded from further analysis as they did not qualify for further investigation.

Forty-nine percent (24/49) of CrAg-positive children had documented meningitis symptoms at the time of the reflex CrAg test. The most common symptoms were headache and vomiting in 14 children. Other symptoms included fever (n=8), photophobia (n=7), seizures (n=6) and confusion (n=6). Only two CrAg-positive patients had headache as the sole symptom. Ten (20%) CrAg-positive patients were documented as asymptomatic, and for 15 (31%), there was no documentation of symptoms. In comparison, three CrAg-negative patients had a headache at the time of the screening test with vomiting (n=3), fever (n=2) and seizures (n=1) documented.

75% (18/24) of the symptomatic CrAg-positive children had a documented abnormal neurological examination including neck stiffness (n=8), focal neurology (n=6) and altered mental status (n=3). No asymptomatic child or child without documented symptoms had an abnormal neurological examination in their records. 6% (3/51) CrAg-negative children had abnormal neurological signs and were diagnosed with disseminated tuberculosis (TB), stroke or HIV encephalopathy.

### Clinical management

The clinical management of the 49 CrAg-positive children eligible for further investigations are summarized in Table 1. In total, 65% (32/49) of CrAg-positive children had a documented lumbar puncture (LP), including 79% (19/24) of those with symptoms. The reason for not performing an LP was usually not documented. One child was treated empirically with amphotericin B, ceftriaxone, acyclovir and TB therapy without an LP because of possible raised intracranial pressure.

Thirty-three percent (16/49) of CrAg-positive children were diagnosed with CM at the time of the reflex test. All but one were symptomatic. 58% (11/19) of symptomatic children who underwent a LP had a CSF positive for either India Ink stain or CrAg test. Three children with severe symptoms of meningitis were treated empirically for CM as a LP was not performed (n=2, of which one had a positive blood culture for *Cryptococcus neoformans*), or with negative findings on the CSF (n=1). All patients with neck stiffness were diagnosed with CM, but some with focal neurology and altered mental status had alternative diagnoses including TB meningitis and severe gastroenteritis.

Only 40% (4/10) of asymptomatic children had a documented LP and none had evidence of *Cryptococcus* in their CSF. Nine out of 15 children with no documented symptoms had a LP and one had a CSF positive for *Cryptococcus* (India ink, CSF CrAg and CSF culture positive).

### Treatment

All children who were diagnosed with CM (n=16) were prescribed antifungals except one who died before the blood culture result was known. Only 20% (3/15) received a WHO-preferred regimen of amphotericin B and flucytosine followed by fluconazole, the others received only amphotericin B and fluconazole. Reported adverse events of the antifungal treatment included hypocalcemia (n=4), anemia (n=2) and acute kidney injury (n=1). Pre-emptive fluconazole was prescribed to 64% (21/33) of CrAg-positive children without a diagnosis of CM.

59% (29/49) of CrAg-positive and 75% (38/51) of CrAg-negative patients were documented to be on ART at the time of CrAg screening (p = 0.10). Fifteen CrAg-positive and 10 CrAg-negative children were documented to have initiated ART for the first time after the screening test, leaving four CrAg-positive children not on ART and for four (1 CrAg-positive and 3 CrAg-negative), the information was lacking.

### Outcomes

Follow-up mortality data was missing for 15 CrAg-positive and 15 CrAg-negative children. Seven (7/36; 19%) CrAg-positive children died in the six months following their reflex CrAg test. 21% (5/24) of symptomatic CrAg-positive children died, four of which were in-hospital CM deaths. One asymptomatic child died of CM 3 months after the positive CrAg test, and one child with no documented symptoms and a negative LP at the time of the CrAg test died of renal insufficiency three months later. Mortality in CrAg negative children was lower than CrAg-positive children (3% (1/36) vs. 19% (7/36); p=0.02) at six months.

Ten CrAg-positive children (10/49; 20%) had an episode of symptomatic CM in the 12-months following the CrAg test compared to none in those who tested negative (p=0.001). Two of these 10 children were from the symptomatic group and had a second episode of CM in the follow-up period. The remaining eight were from the asymptomatic/ no documented symptoms group who had their first episode of CM in the 12 months following the reflex CrAg test. Therefore, excluding eight children with missing data, the likelihood of a child with no documented symptoms developing symptomatic CM in the 12-months following a positive CrAg test was 47% (8/17). Only one of these children died.

Five percent (1/19) of children pre-emptively treated with fluconazole subsequently presented with CM, compared to 58% (7/12) of those with no documented fluconazole prescription (p=0.002; absolute risk reduction 53% (95% CI 23%-83%); number needed to treat 1.9 (95% CI 1.2-4.3)). The child who died of CM three months after a reflex CrAg test had a positive result, was documented as asymptomatic, did not have an LP and was not prescribed pre-emptive fluconazole.

### Children less than 10 years old

Seven (14%) CrAg-positive children (and seven matched CrAg-negative controls) included in this study were <10 years old, with a median age of 6 years (range, 2-8) and male to female ratio 1:1.3. Their CD4 counts ranged from 6 to 87 cells/µL and CD4 percentages from 0.52% to 8.15%. Clinical presentation (43% symptomatic, 29% asymptomatic) and mortality (15%) was comparable to the overall population. Three received treatment for CM and 3 received pre-emptive fluconazole. One 5-year-old child who was not treated pre-emptively developed CM 11 months later.

## Discussion

This large study clearly shows that prevalence of antigenemia in South African children is similar to the 5.8% published in adults with CD4 counts <100 cells/µl. The prevalence in children 0-17 years of age with a CD4 count of <100 cells/µl was 4.7%. In children 0-9 years of age, the prevalence was 3.5%. KwaZulu-Natal Province had the highest CrAg prevalence, with 7.1% among 0–17-year-olds and 4.6% among 0–9-year-olds. This province has the highest HIV prevalence in South Africa and has the highest provincial CrAg prevalence in adults at 7.6%.^21, 27, 28^ A greater total number of children with a CD4 count of <100 cells/µl in Kwazulu-Natal Province would not fully explain why a higher percentage of them tested CrAg positive. One explanation could be that the median CD4 count of children tested in this province is lower, with a corresponding higher risk of cryptococcal disease reactivation. Another could be higher environmental exposure. Our calculated prevalence in children in KwaZulu-Natal is higher than previously published. This may be because our study included CrAg results with no gender or age specified (provided that a date of birth was supplied) which were excluded by Zuma *et al*.^22^ CrAg prevalence in South African children is lower than that reported from Cameroon, but adult CrAg prevalence was also higher in Cameroon at 7.5%.^23^

Our results do raise the question of why children living with HIV and with CD4 counts <100 cells/µl are not included in the recommendation for CrAg screening. As shown in our study, children <10 years of age and CD4 counts <100 cells/µL are equally at risk to develop and succumb to CM as older children.

Half of the 49 CrAg-positive children were already symptomatic when the reflex CrAg test was performed and of those, two-thirds were diagnosed with CM. The additional benefit of the reflex blood CrAg test in symptomatic children is the facilitation of an earlier diagnosis of CM in children in a clinical scenario with a low index of suspicion. Many clinicians in South Africa do not routinely request a CSF CrAg test in immunosuppressed children presenting with suspected meningitis due to the belief that children are not at risk of CM, despite South African HIV Clinicians Society guidelines being updated to recommend the test in adults, adolescents and children less than 5 years old.^20^ Our data provides evidence that CM should be considered in the differential diagnosis of children clinically presenting with meningitis.

Of the CrAg-positive children, only 65% had a documented LP and only 64% of eligible children received pre-emptive fluconazole. Pre-emptive fluconazole led to an absolute risk reduction of subsequent CM of 53%. Including only those with documented follow-up, the six-month mortality of CrAg-positive children was 19% (compared to 3% in matched CrAg-negative children). The number of children with no documented LP and those not receiving pre-emptive fluconazole argues for the development and implementation of CrAg screening and management guidelines in this age group.

The main limitation of this study is the small number of children included in the medical record review. Retrospective studies using paper medical records in South Africa are extremely challenging due to the complexity of the approval processes and the heavy workload facing medical record departments, which are often operating under resource constraints. Even despite multiple and extensive visits to each facility, we could not include more children in the medical record review. Nevertheless, the results are very valuable as there is a paucity of clinical data on pediatric cryptococcosis. When calculating CrAg prevalence, results with no date of birth were excluded. This may lead to an overestimation of prevalence but was done to ensure no adults were included. In addition, some of the CrAg-positive children might have had CM prior to 2017, with a residual positive CrAg test result.

This study only included children with an absolute CD4 count <100 cells/µL as this is the threshold for reflex CrAg testing of adults and adolescents in South Africa. Fewer CD4 tests are now being performed in children than previously due to a transition to using viral load for monitoring and cost concerns.^3^ Moreover, using a <100 cells/µl threshold does not capture all children living with HIV with stage three immunosuppression at risk of opportunistic infections, as the absolute CD4 count which defines stage three is higher in children under six years old. Further investigation is required to determine whether pediatric-specific thresholds are required, for example using CD4 percentages for children 5-9 years old, so vulnerable children are not excluded from screening using adult reference values.

In conclusion, CrAg prevalence in children with an absolute CD4 count of <100 cells/µL is comparable to that in adults. Implementation of CrAg-screening guidelines in the pediatric population should be extended to include children <10 yrs of age to improve outcomes.

## Figures and Tables

**Table 1:** Clinical presentation, management and outcome of children (0-17 years) with a positive or negative cryptococcal antigen reflex test included in the medical record review.

		CrAg positive reflex screening test			CrAg negative
		Symptomatic N=24	Asymptomatic N=10	Symptoms not documented in records N=15	N=51
Age, years (median, IQR)		14 (13-15)	14 (13-16)	15 (12.5-16)	14 (12.5-16)
Males		13 (54%)	6 (60%)	8 (53%)	28 (55%)
CD4 T-cells (%)		2.6 (1.6-4.0)	1.6 (0.7-3.6)	1.3 (0.8-2.9)	2.0 (0.9-3.5)
CD4 T-cells (cells/μl)		15 (10-37)	14 (6-47)	12 (5-18)	21 (7-41)
On ARV		12 (50%)	10 (100%)	7 (47%)	38 (75%)
Abnormal neurologic examination		18 (75%)	0 (0%)	0 (0%)	3 (9%)
CSF positive for Cryptococcus*		11/19 (58%)	0/4 (0%)	1/9 (11%)	0
CM diagnosed^$^		15* (63%)	0 (0%)	1 (7%)	0 (0%)
Therapy	Targeted treatment	14/15@ (93%)	NA	1/1 (100%)	NA
	Pre-emptive fluconazole	6/9 (67%)	5/10 (50%)	10/14 (71%)	NA
CM within 12 months		2 (8%)	4 (40%)	4 (27%)	0
6-month mortality		5 (21%)	1 (10%)	1 (7%)	1 (2%)

ARV: antiretroviral therapy, CSF: cerebrospinal fluid, CM: cryptococcal meningitis, CrAg: cryptococcal antigen, NA: not applicable

* Indian Ink or cryptococcal antigen positive

$ Includes empiric diagnoses and when LP not performed but blood culture positive for *Cryptococcus* sp.

@ One child died before diagnosis of cryptococcal meningitis (CM) known
